# Clinical audit: crisis in the communication of abnormal blood test results in the wards

**DOI:** 10.1186/1753-6561-7-S1-O2

**Published:** 2013-01-30

**Authors:** Razeen Hassan, K Anpalagan, D Murphy, I Callanan

**Affiliations:** 1 Royal College of Surgeons in Ireland, Dublin, Ireland; 2 St. Vincent’s University Hospital, Dublin, Ireland

## Background

Strict adherence to the established standards^1^ is expected in the communication of abnormal laboratory results between the laboratory and the ward. Upon receiving abnormal laboratory results, the laboratory personnel will phone and inform the ward about the results. The receiver will take the necessary actions and then transfer the abnormal results into the patient’s charts. In a three-week audit conducted, we compared the compliance of all wards in SVUH in acting upon and transferring the abnormal laboratory results into the charts against the standards set by Joint Commission International (JCI), whereby 100% compliance towards the process is expected.

## Methods

Retrospective, random data collection of 100 abnormal laboratory results (50-biochemistry and 50-haematology) were compared to the details found in the corresponding patients charts using a data collection form consisting of three questions.

## Results

Out of 100 cases, 39% of abnormal laboratory results were not written down, the team was not told and there was no action taken (all YES). In 32% of cases, the results were written down, the team was told and action was taken (all NO). In 27% of cases, the results were not written down and there was no indication that team was told but action was taken. In 2% of cases, the results were written down and team was told but action was not taken. Out of 13 wards, 2 reported high rates of compliance (3/3 and 6/8 cases with all YES) and 2 exhibited high rates of non-compliance (6/13 and 6/7 cases with all NO). One ward quoted a ‘different nursing care plan’ for their non-compliance.

## Conclusions

Our study discovered a worryingly high non-compliance rate among medical ward staff towards the process of transferring the abnormal laboratory results into patients’ charts, while full compliance rate is low at 32%. There are inconsistencies between the different nursing care plans and the delay in the timely follow-up of patients’ abnormal laboratory results raises a safety concern. We propose the use of detachable stickers that could be peeled off and pasted into patients’ charts to increase compliance.

